# NADPH Oxidase and Epidermal Growth Factor Receptor Are Promising Targets of Phytochemicals for Ultraviolet-Induced Skin Carcinogenesis

**DOI:** 10.3390/antiox10121909

**Published:** 2021-11-29

**Authors:** Min Jeong Kim, Su Jeong Ha, Bo Ram So, Chang-Kil Kim, Kyung-Min Kim, Sung Keun Jung

**Affiliations:** 1 School of Food Science and Biotechnology, Kyungpook National University, Daegu 41566, Korea; minjeongkim@knu.ac.kr (M.J.K.); boram26@knu.ac.kr (B.R.S.); 2 Department of Agricultural Biotechnology, Seoul National University, Seoul 08826, Korea; igiihsj@snu.ac.kr; 3 Department of Horticultural Science, Kyungpook National University, Daegu 41566, Korea; ckkim@knu.ac.kr; 4 Division of Plant Biosciences, School of Applied Biosciences, College of Agriculture and Life Science, Kyungpook National University, Daegu 41566, Korea; 5 Institute of Agricultural Science & Technology, Kyungpook National University, Daegu 41566, Korea

**Keywords:** nicotinamide adenine dinucleotide phosphate oxidase (NADPH oxidase), epidermal growth factor receptor (EGFR), protein tyrosine phosphatase κ (PTPκ), reactive oxygen species (ROS)

## Abstract

The skin acts as the primary defense organ that protects the body from the external environment. Skin cancer is one of the most common cancers in the world. Skin carcinogenesis is usually caused by cell degeneration due to exposure to ultraviolet (UV) radiation, which causes changes in various signaling networks, disrupting the homeostasis of single skin cells. In this review, we summarize the roles of nicotinamide adenine dinucleotide phosphate oxidase (NOX) and epidermal growth factor receptor (EGFR) in UV-induced skin carcinogenesis. Furthermore, we describe the crosstalk that exists between NOX, EGFR, and protein tyrosine phosphatase κ and its oncogenic downstream signaling pathways. Chemoprevention is the use of chemical compounds to recover the healthy status of the skin or delay cancer development. Current evidence from in vitro and in vivo studies on chemopreventive phytochemicals that target NOX, EGFR, or both, as major regulators of skin carcinogenesis will also be discussed.

## 1. Introduction

Based on recent cancer statistics, 10 million people died of skin cancer (9.9 million excluding nonmelanoma skin cancers [NMSCs]) in 2020 [1]. The most frequently diagnosed types of skin cancers, collectively known as NMSCs, are basal cell carcinoma (BCC) and squamous cell carcinoma (SCC) [2]. While NMSC is the most frequently detected skin malignancy, which accounts for 40% of all cancers diagnosed in the United States [3], malignant melanomas are detected in only 4% of all skin cancers [4]. BCCs and SCCs develop from within the basal layer of the epidermis. While BCCs have slow growth and rarely metastasize, SCCs are more invasive and metastasize 10% of the time [5,6].

Ultraviolet (UV) light, inevitably met in daily environments and substantially affecting skin homeostasis, is one of the factors that can harm skin health. Epidemiological studies have proven that solar UV irradiation is the most important environmental carcinogen contributing to skin cancer development [7]. UVC is absorbed in the stratospheric ozone. By contrast, UVB (1–10%) and UVA (90–99%) reach the surface of the earth and ultimately cause DNA damage, erythema, sunburn, immunosuppression, and skin cancer in humans [8]. The UVA irradiation to which we are exposed appears to be weakly carcinogenic and causes photoaging and wrinkling of the skin. On the other hand, UVB is recognized as a complete carcinogen relevant to human skin cancer [9].

Prolonged exposure to UV impairs skin homeostasis and stimulates responses to environmental changes, including the production of reactive oxygen species (ROS). These responses lead to the activation of multiple signaling cascades, which switch on the expression of oncogenic and tumor suppressor genes. A number of studies have proven that antioxidants contribute to the prevention of skin carcinogenesis. However, a part of phytochemicals with low antioxidant capacity demonstrates relatively high chemopreventive effects. Furthermore, several compounds, including vitamin D and lycopene, can act as pro-oxidants. We reported in various studies that phytochemicals can prevent cancer by acting as inhibitors of the oncogenic molecules, Fyn, Src, aryl hydrocarbon receptor (AhR), phosphoinositide 3-kinase (PI3K), Raf, and extracellular signal-regulated kinase (ERK)2 both in vitro and in vivo. In this review, we introduce nicotinamide adenine dinucleotide phosphate (NADPH) oxidase and epidermal growth factor receptor (EGFR) as molecular targets that can complement antioxidant activity and effectively inhibit skin cancer via the regulation of various signaling networks. In addition, based on recent research [10], we highlight the function of protein tyrosine phosphatase κ (PTPκ) as a mediator of EGFR signal activation by NADPH oxidase (NOX).

The sentence, “prevention is better than a cure” highlights the importance of prevention. Many scientists and laboratories are working to develop chemopreventive materials. However, materials that have proven to be effective in counteracting diseases, such as cancer, are still needed. By summarizing the results of our research and that of other scientists, we aim to introduce materials that could prevent or control skin cancer and skin diseases through the regulation of NOX and EGFR.

## 2. Skin Carcinogenesis and Chemoprevention

Chemoprevention is defined as the use of a drug or appropriate treatment to delay the onset of cancer or to return cancer to normal [11]. It takes over a decade for cancer to develop [12,13]. Skin cancer develops in multiple stages, which is similarly observed in other cancers. Specifically, in skin carcinogenesis, there are three stages: initiation, promotion, and progression. Acute UV exposure can initiate skin carcinogenesis, which is characterized by DNA mutations and abnormal gene activation or suppression. Promotion is the clonal expansion of damaged cells via aberrant signaling pathways, which takes place over many years. It involves uncontrolled tumor growth that can invade and metastasize to secondary organs [14,15,16]. Because initiation is an irreversible step lasting only for a short duration (seconds or minutes), the prevention of initiation is difficult to achieve using chemopreventive agents. However, targeting the promotion stage has attracted significant interest because of its characteristic reversibility and long duration (a decade) [15,16]. During the promotion stage, mutations of tumor suppressor genes, including p53, and hyperactivation of inflammatory transcription factors, such as activator protein 1 (AP-1) and nuclear factor kappa-light-chain-enhancer of activated B cells (NF-κB), can occur. Subsequently, abnormal production of the inflammatory enzyme, cyclooxygenase-2 (COX-2), and proinflammatory cytokines, interleukin (IL)-1β, IL-6, and transforming growth factor β (TGFβ), are some of the other features of promotion. Therefore, a vast of research aimed at the development of chemopreventive phytochemicals has focused on targeting oncogenic signaling molecules.

## 3. Role of Dinucleotide Phosphate Oxidases (NOXs) and Their Downstream Signaling Pathways in UV Radiation-Induced Skin Carcinogenesis

Excessive cellular ROS play a critical role in skin carcinogenesis by inducing genotoxicity via DNA damage, which causes inactivation of tumor suppressor genes and activation of proto-oncogenes. ROS also promote skin carcinogenesis by non-genotoxicity through hyperactivation of oncogenic signaling pathways [17]. NOXs, which are membrane-bound enzymes, respond to UVB and are essential for cellular ROS production [18]. NOXs consist of a catalytic subunit (NOX1 through NOX5, dual oxidase (Duox)1, and Duox2), p22phox, p47phox, p67phox, and the small guanosine triphosphatase, Rac1 [19]. NOX1–NOX3 must bind to their corresponding subunits in order to become activated; however, NOX4 is constitutively active, while NOX5 and the Duox enzymes are calcium-dependent via EF-hands [20,21]. NOX1, 2, 4, and 5 mRNA and NOX1, 2, and four proteins have been identified and are the principal producers of ROS in immortalized human keratinocyte HaCaT cells [22,23].

**Figure 1:**
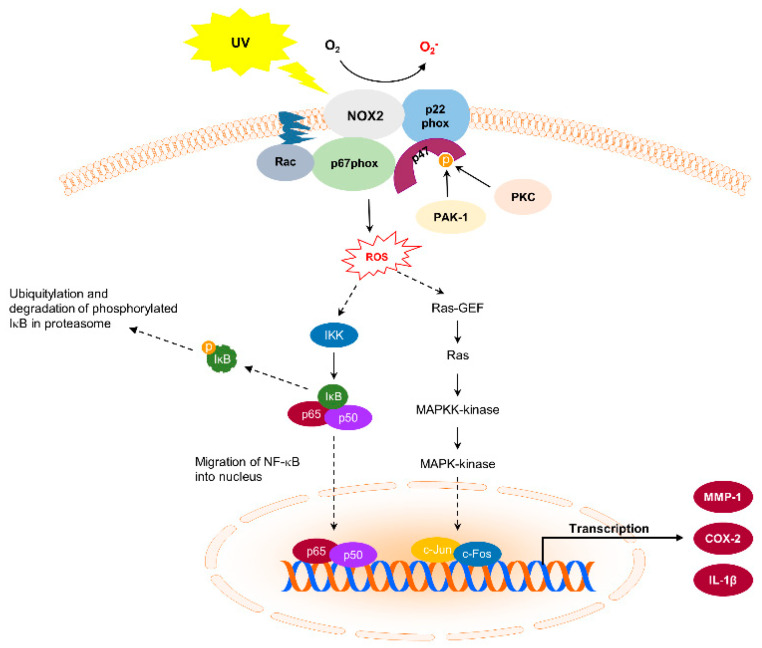
Nicotinamide adenine dinucleotide phosphate oxidase (NOX) and its downstream signaling pathways in ultraviolet (UV)-induced skin carcinogenesis.

Generally, UVB irradiation triggers a DNA damage response network that includes cell apoptosis through excessive cell cycle progression and damage to DNA repair machinery [24]. A specific type of UVB-mediated DNA damage increases the occurrence of cyclobutane pyrimidine dimers [25,26,27]. Therefore, NOX-mediated malfunction of nucleotide excision repair, an essential proofreading system to repair UVB-induced DNA damage, plays a crucial role in skin carcinogenesis [25,28]. A clinical study proposed that *N*-acetyl cysteine (NAC), a NOX inhibitor, could prevent pro-oncogenic oxidative stress in nevi and alleviate the risk of long-term melanoma when taken by patients [29]. However, a phase II randomized placebo-controlled trial reported that a single oral dose of NAC did not show a significant protective effect on UV-induced nevi due to oxidative stress [30]. The development of skin cancer is observable in SKH-1 hairless mice that are subjected to chronic UVB exposure. However, topical treatment with apocynin, which inhibits NOX activity, prevents two stages of UVB-induced skin carcinogenesis in these mice [31]. Furthermore, direct inhibition of NOX1 with a specific peptide inhibitor, lnhNOX1, has been shown to suppress UVB-induced bursts of ROS production, increase nucleotide excision repair efficiency, decrease apoptosis in HaCaT cells, and decrease skin carcinogenesis in Xpc wild-type mice [24]. These results indicate that NOX plays a critical role in UV-induced skin carcinogenesis and could be a promising target for the generation of chemopreventive materials (Figure 1).

UV irradiation and excessive ROS production cause mutations in the protein patched homolog 1 (PTCH) gene, encoding a membrane receptor, which results in the activation of the Hedgehog signaling pathway involved in the pathogenesis of BCC [32]. Once DNA is damaged by acute UV irradiation, p53 makes the DNA repair protein for DNA replication, repairs and regulates the cell cycle, or activates apoptosis-signaling pathways if the DNA damage is irreversible [33,34]. In addition, prolonged UV exposure induces the accumulation of mutations in phosphatase and tensin homolog (PTEN), a tumor suppressor gene that regulates the PI3K/Akt signaling pathway through its phosphatase activity. The results in low expression levels of PTEN in SCC [35,36]. Therefore, damage to p53 and PTEN, due to ROS makes it hard to repair DNA damage caused by UV radiation, ultimately leading to skin cancer. Based on the use of pharmaceutical inhibitors for NOX, Ha et al. observed that NAC suppressed UVB-induced phosphorylation of mitogen-activated protein kinases (MAPKs), Src, and EGFR in HaCaT cells [10]. Therefore, in addition to directly scavenging ROS using antioxidants, modulating EGFR, PI3K, Src, and MAPK activity, such as by phosphorylation, would be a remarkable strategy to prevent UV-induced skin damage and skin cancer.

## 4. Role of Epidermal Growth Factor Receptor (EGFR) and Its Downstream Signaling Pathways in UV-Induced Skin Carcinogenesis

The epidermal growth factor (EGF) receptors include: ErbB-1 (HER1), ErbB-2 (HER2), ErbB3 (HER3), and ErbB4 (HER4) [37]. These receptors are composed of extracellular ligand-binding, transmembrane, and intracellular tyrosine kinase domains [38,39]. Because hyperactivity and various mutations of EGFR contribute to the development of cancer and drug resistance, many anticancer drugs targeting EGFR are being studied [40,41,42,43]. EGFR homodimers and heterodimers are formed when a soluble ligand binds to the receptor’s ectodomain. This step is essential for the activation of tyrosine kinase receptors, such as EGFR, via phosphorylation of the C-terminal tail. We have confirmed that UV irradiation upregulates EGFR phosphorylation at Tyr1068, Tyr1045, and Tyr845 in HaCaT cells [10,44].

**Figure 2:**
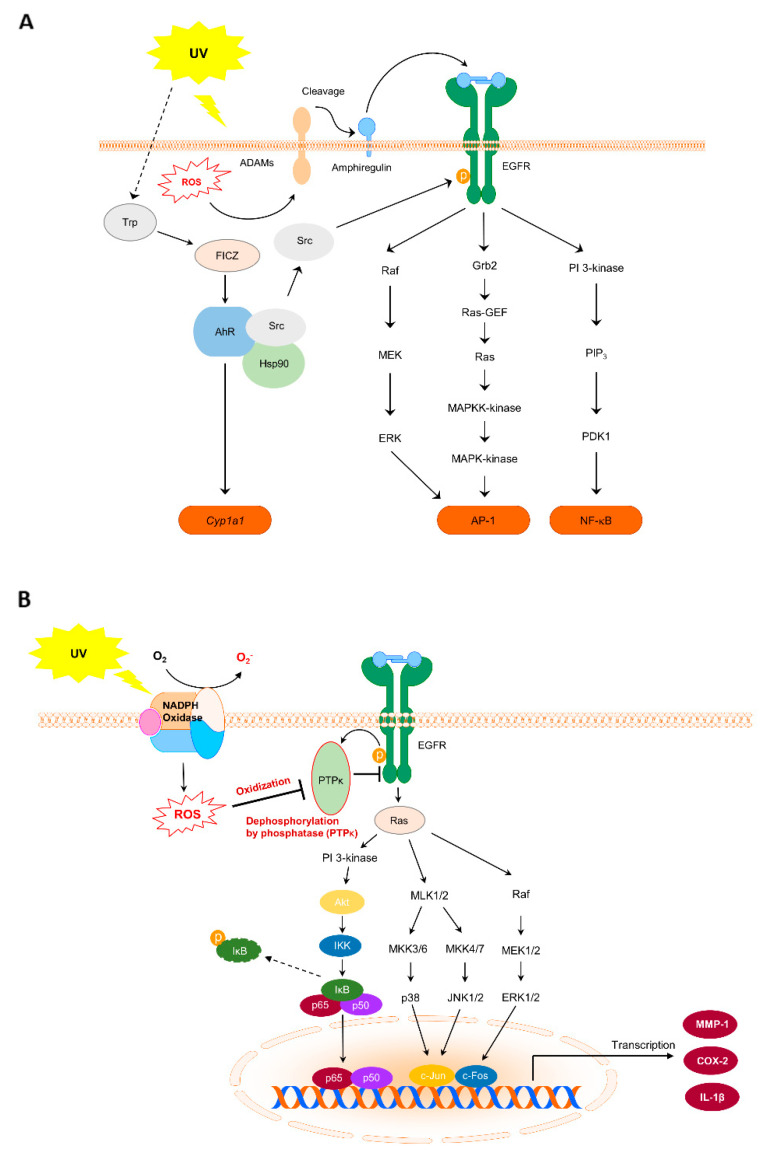
Epidermal growth factor receptor (EGFR) and its downstream signaling pathways, and the possible mechanisms of UV radiation-induced skin carcinogenesis. (**A**) EGFR and its downstream signaling pathways in UV-induced skin carcinogenesis. (**B**) NOX and PTPκ signaling pathways in UV-induced skin carcinogenesis.

As a principle of EGFR activation after UV exposure, abnormal ROS production activates a disintegrin and metalloproteinase (ADAM), which performs cleavage to form the EGFR ligand, pro-amphiregulin, at the cell membrane [45]. Free amphiregulin then binds to EGFR and triggers a conformational change of the receptor. EGFR subsequently activates itself through dimerization. We confirmed that amphiregulin expression increases in response to UV irradiation [10] (Figure 2A). Activated EGFR regulates multiple signaling pathways, including p38, c-Jun NH2-terminal kinase (JNK), ERK, PI3K/Akt, and protein kinase C. PI3K/Akt are phosphorylated following UV irradiation in HaCaT cells. Interestingly, UV irradiation brings about the dissociation of AhR and Src complexes, enabling Src to phosphorylate EGFR at Tyr845. Consequently, this phosphorylation increases cyp1a1 mRNA and COX-2 protein expression in HaCaT cells [44]. In short, UV-induced EGFR activation accelerates multiple signaling pathways involved in skin carcinogenesis. Agents with the ability to modulate these signaling cascades could serve as promising chemopreventive materials.

## 5. Role of Protein Tyrosine Phosphatase κ (PTPκ) in NOX and EGFR Signal Transmission in Skin Cancer

An early study suggested that hydrogen peroxide induces EGFR activity via the inhibition of protein tyrosine phosphatase (PTP). This was demonstrated by the addition of catalase, an enzyme that uses hydrogen peroxide as a substrate, which inhibited EGF-induced tyrosine phosphorylation of phospholipase Cγ1 (PLCγ1), a physiological substrate of the EGFR [46]. Furthermore, several studies have proven that ROS produced by NOX regulates EGFR. NOX4 mediates the oxidation and inactivation of PTP1B in the endoplasmic reticulum (ER) and switches on the EGFR signaling pathways [47]. In addition, activation of NOX by active mutation of TRA4 induces oxidative damage of focal contact phosphatase PTP-PEST [48]. David et al. described the relationship of PTP and EGFR with NOX, the signaling network of EGFR, and the interrelationships within the redox system [49,50]. A recent study revealed the direct relationship between UVB-induced ROS accumulation and the inactivation of PTPκ, which is a phosphatase specific for EGFR [51]. UVB irradiation increases ROS production and NOX activity. It also downregulates PTPκ activity, subsequently upregulating MAPKK and MAPK signaling cascades, and MMP-1 and COX-2 expression in HaCaT cells [10]. Exposure to chronic UVB irradiation in SKH-1 hairless mice led to the development of skin cancer in a two-stage skin carcinogenesis model [10]. Therefore, the interaction of NOX, EGFR, and PTPκ is a crucial factor in skin carcinogenesis. Controlling this interaction is a core approach to prevent UV-induced skin cancer (Figure 2B).

## 6. Botanical Extracts and Phytochemicals Preventive in UV-Induced Skin Damage via the Regulation of NOX

UVB induces NOX expression in skin tissue, thereby increasing oxidant production. NOX can be assessed both directly, by its activity, and indirectly, by measuring catalase, superoxide dismutase activities, and glutathione depletion (Table 1). To assess whether or not botanical extracts or phytochemicals altered NOX activity, previous studies measured NOX activity, NOX gp91phox mRNA expression, and p47phox translocation.

Eupafolin, 6-methoxy 5,7,3′,4′-tetrahydroxyflavone, is a vital component of the methanolic extract of *Phyla nodiflora* [52]. Human dermal fibroblasts (Hs68) treated with eupafolin in vitro showed reduced LPS-induced ROS generation via deterioration of p47*^phox^* activity and decrease translocation of p47*^phox^* from the cytosol to the plasma membrane. The inhibition of LPS-induced COX-2 expression was found to be mediated by transfection with scrambled siRNAs targeting the cytosolic regulatory subunit, p47*^phox^*, and membrane catalytic subunit, Nox2 (gp91*^phox^*). The COX-2 gene promoter contains consensus-binding sites for both AP-1 and NF-κB; however, eupafolin only reduces the LPS-induced increase in AP-1 activity through the phosphorylation of MAPK. In an in vivo study, male C57BL6 mice were injected subcutaneously with LPS for 24 h following a 30 min pre-treatment with eupafolin (administered intraperitoneally). The results demonstrated that eupafolin induced lower COX-2 expression in the mouse dermis than LPS alone [52]. Overall, the LPS-induced inflammatory response in the skin can be attenuated by inhibiting the Nox2/p47*^phox^*/ROS/JNK/AP-1-dependent pathway.

*Trans*-chalcone [53], 1,3-diphenyl-2-propen-1-one, is the biphenolic precursor of flavonoids in plants. An in vivo experiment was performed using male, hairless mice (HRS/J) treated intraperitoneally with *trans*-chalcone for 1 h before UV irradiation. *Trans*-chalcone reduced UVB-induced excessive generation of superoxide anion and gp91*^phox^* mRNA overexpression in these mice. The relationship between acute inflammation and oxidative stress, including the critical roles of NOX2 and ROS as secondary messengers in pro-inflammatory signaling cascades, has been previously described. *Trans*-chalcone reduces proinflammatory Th2 and Th17 cytokines. In addition, *Trans*-chalcone diminishes epidermal thickness and the number of sunburnt cells in the skin of hairless mice exposed to UV radiation. *Pimenta pseudocaryophyllus* belongs to the Myrtaceae family and contains flavonoids and other polyphenolic compounds [57]. *P. pseudocaryophyllus* ethanolic extract (PPE) inhibited UVB-mediated increase in skin edema, gp91*^phox^* mRNA overexpression, and IL-1β production. It also increased IL-10 levels in the same model experiment relative to *trans*-chalcone. This compound and extract were suggested to prevent skin diseases, such as oxidative stress and skin inflammation.

Apocynin, a known NOX inhibitor, is an active compound derived from the roots of the medicinal plant *Picrorhiza kurroa* [31]. In vitro, UVB-irradiated epidermal JB6 P^+^ cells from mouse skin were used to study skin carcinogenesis. Pretreatment with apocynin suppressed NOX activity, phosphorylation of MAPK and Akt signaling, and COX-2 activity compared with UVB- irradiated JB6 P^+^ cells. Development of skin tumors was observed within 21 weeks by exposure to UVB, whereas apocynin attenuated UVB-induced tumors in female SKH-1 hairless mice. 

Syringic acid is a component of the açaí palm (*Euterpe oleracea*), which contains hydroxybenzoic acid derivatives [10]. UVB-induced NOX activity and phosphorylation of MAPKs, MAPKKs, B-Raf, Akt, and Src were inhibited by syringic acid treatment, in addition to a reduction in the excessive expression of MMP-1 and COX-2 in HaCaT cells. Syringic acid inhibited COX-2 and MMP-13 expression, mouse epidermal thickness, and the number of tumors per mouse in SKH-1 hairless mice exposed to UVB (0.2 J/cm^2^) irradiation 3 days/week for 22 weeks.

Apocynin and syringic acid have been shown to inhibit NOX and AP-1 or NF-κB promoter activity, preventing skin carcinogenesis. Thus, phytochemicals can be used to target UV light-induced activation of transcription factors, such as NF-κB and AP-1 in skin carcinogenesis.

## 7. Botanical Extracts and Phytochemicals Preventive in UV-Induced Skin Damage via the Regulation of EGFR

Because EGFR has a central role in the growth and survival of cancer cells, many materials have been developed to target EGFR directly. However, the long-term use of EGFR-targeting drugs, such as gefitinib and erlotinib, was accompanied by adverse drug reactions, particularly in the skin. Furthermore, these drugs ultimately failed in female Asian patients with non-small cell lung cancer [58]. Therefore, we investigated the effects of extracts or phytochemicals in the control of upstream or downstream signaling of EGFR, rather than focusing on its direct regulation.

Several studies have explored the effects of nutraceuticals on UV-stimulated skin, in vitro and in vivo, concerning EGFR phosphorylation. However, ERBB activation, dysregulation of amphiregulin and AhR, and excessive phosphorylation of Src can all be targeted to prevent skin diseases. Therefore, various materials can inhibit skin inflammation or cancer by reducing factors linked to EGFR activation (Table 2).

The AhR signaling pathway is one of the major UVB-induced signaling pathways, and AhR mediates COX-2 overexpression. We demonstrated that 1,8-cineole, a monoterpene cyclic ether, inhibits c-Src-dependent EGFR activation induced by UVB, thereby suppressing the RAF-MEK1/2-ERK1/2 signaling pathway and cyp1a1 mRNA expression, a target gene of activated AhR [44]. This was demonstrated by the knockdown of AhR, which suppresses COX-2 expression in HaCaT cells. 1,8-cineole also reduced UVB-induced epidermal thickness and skin tumorigenesis in hairless SKH-1 mice. Overall, UVB-induced skin carcinogenesis could be suppressed by directly targeting AhR.

Oi et al. demonstrated via in silico modeling that taxifolin, an ATP-competitive inhibitor, can bind to the ATP-binding pocket of EGFR (Lys 721, Met 769, and Asp 831) and suppress UVB and solar UV-induced carcinogenesis [59]. They also showed that taxifolin regulates COX-2 through EGFR inhibition. In addition, TGa cellulose nanocrystal [60], carnosic acid [61], and cyanidin-3-*O*-glucoside [63] decreased downstream kinases and Akt or MAPK signaling pathways by reducing UVB-induced phosphorylation of EGFR.

In a recent study, Ha et al. identified that the phosphorylation of EGFR and PTPκ, a regulator of EGFR activation, could be modulated by syringic acid [10]. Notably, oxidized PTPκ is a factor involved in UVB-induced EGFR activation. Among numerous observations, they noted that syringic acid inhibited NOX activity, mainly by its Nox/PTPκ/EGFR axis-driven skin carcinogenic activity [10]. Thus, they suggested that nutraceuticals could regulate a link between the impairment of PTPκ and its rescue. Moreover, it was confirmed that the inhibition of PTPκ oxidation suppressed downstream signaling factors, including Ras, Akt, and MAPK.

The appearance of wrinkles coinciding with MMP-13 and COX-2 expression has also been observed using models of UVB-mediated skin inflammation and photoaging in SKH-1 hairless mice. Both *Rhus javanica* [64] and *Curcuma zedoaria* [65] extracts, administered orally, improved the outcomes of these models. *Curcuma zedoaria* extract reduces MMP-1 expression, phosphorylation of JNK1/2, MKK3/6/p38, B-Raf/MEK1/2/ERK1/2, Src, EGFR (Y1068 and Y1045), and Akt after UVB irradiation in HaCaT cells. Furthermore, *R. javanica* extract demonstrates the same reduction effect as *C. zedoaria* extract and inhibits the phosphorylation of PKD/PKC_μ_ and Tyr. It also significantly inhibits EGFR kinase activity.

Curcumin, a dietary polyphenol, is the main component of turmeric and is extracted from the East Indian plant, *Curcuma longa*. According to Hosur et al., curcumin treatment inhibited an increase in ear swelling, ERK phosphorylation, and inflammatory cytokine production in a dermatitis model established by dosing female BALB/c mice with 2, 4, 6-trinitrochlorobenzene (TNCB) [66]. Furthermore, amphiregulin gene overexpression was also attenuated by curcumin treatment in the TNCB-challenged ear. Moreover, Capalbo et al. reported preclinical evidence that while conventional EGFR-targeted treatments, such as cetuximab, show resistance, oral curcumin phospholipid supplements can overcome resistance to anti-EGFR treatment. However, the mechanism by which curcumin interacts with EGFR has not been fully elucidated.

## 8. Conclusions

**Figure 3:**
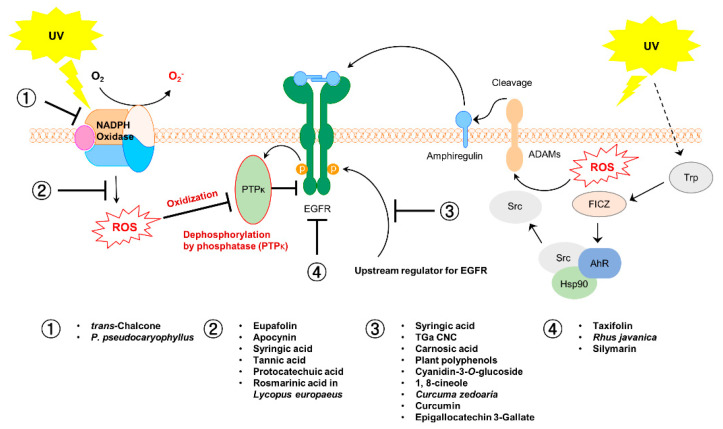
Summary of the mechanism of the effect of botanical extracts and phytochemicals in response to UV irradiation.

**Figure 4:**
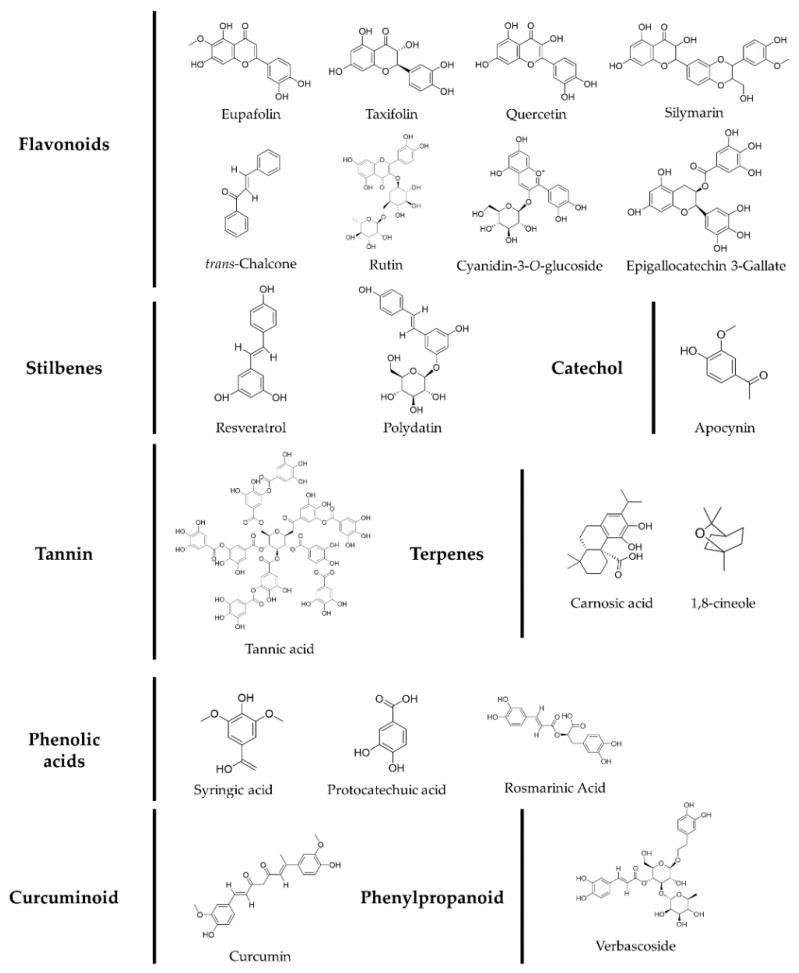
Classification and structures of phytochemicals with regulatory effects on EGFR and NOX activity.

In this paper, we have described NOX, EGFR, and the crosstalk between these two molecules, as crucial regulators of skin carcinogenesis. In addition, we have discussed the phytochemicals and botanical extracts that exert a preventive effect against UV-induced skin carcinogenesis through their regulation of these molecules (Figure 3 and Figure 4).

Various materials have been discussed regarding the prevention of skin cancer via the regulation of NOX and EGFR following activation with UV and subordinate factors; however, research on the direct interplay between the materials and these molecules is incomplete. Therefore, to aid the development of more efficient chemopreventive materials in the future, various aspects should be studied, such as the interactions between materials and their target molecules and their mechanisms of action at the molecular and cellular levels.

## Figures and Tables

**Table 1:** Phytochemicals and their effects on NOX inactivation, enzyme activity, and transcription.

Name	Stimulus	Measurement of NOX Activity	Enzyme Activity or Transcription Factor	Target Disease	Reference
Eupafolin	LPS	NOX activity, p47*^phox^* translocation, COX-2 expression using knockdown of NOX2 (gp91*^phox^*) and p47*^phox^*	Phosphorylation of c-Fos and c-Jun, AP-1 DNA binding activity	Skin inflammation	[52]
*trans*-chalcone	UV source	NOX2 gp91*^phox^* *mRNA* expression	Myeloperoxidase (MPO) activity, Metalloproteinase-9 (MMP-9) activity, GSH depletion, Catalase (CAT) activity	Skin inflammation Oxidative stress	[53]
Apocynin	UVB	NOX activity	AP-1 promoter activity, NF-κB promoter activity	Skin carcinogenesis	[31]
Syringic acid	UVB	NOX activity	Metalloproteinase-1 (MMP-1) expression, AP-1 promoter activity	Skin carcinogenesis	[10]
Tannic acid	UVB	NOX activity	Catalase (CAT) activity, Superoxide dismutase (SOD) activity, GSH depletion, Metalloproteinase-1, 9 (MMP-1, 9) expression	Photoaging	[54]
Protocatechuic acid	UVB	NOX activity	Catalase (CAT) activity, Superoxide dismutase (SOD) activity, GSH depletion, Lipid peroxidation, Metalloproteinase-1, 9 (MMP-1, 9) expression	Oxidative injuries Photoaging	[55]
Rosmarinic acid in *Lycopus europaeus*		NOX2 and NOX4 activity		Photoaging	[56]
*Pimenta pseudocaryophyllus*	UVB	NOX2 gp91*^phox^* mRNA expression	Glutathione reductase mRNA expression, Myeloperoxidase (MPO) activity, Metalloproteinase-9 (MMP-9) activity, GSH depletion, Superoxide anion production, Lipid peroxidation	Skin inflammation Oxidative stress	[57]

**Table 2:** Phytochemicals and their effects on EGFR activation and the phosphorylation of various signaling molecules.

Name		Stimulus	Measurement of EGFR Activity	Dephosphorylation Targets	Target Disease	Reference
Taxifolin		UVB and solar UV	Phosphorylation (T1068) in vitro EGFR kinase assay	P38, JNK, ERK Akt, p70^s6k^, p90RSK, MSK	Skin carcinogenesis	[59]
Syringic acid		UVB	Phosphorylation (T1068, T1045) Activity of the EGFR phosphatase PTP-κ	P38, JNK1/2, ERK1/2 MEK1/2, MKK4/7, MKK3/6, B-Raf, Akt, Src	Skin carcinogenesis	[10]
TGa Cellulose nanocrystal (CNC)		UVB	Phosphorylation (T1068, T1045)	P38, JNK1/2, ERK1/2 MEK1/2, MKK4/7, B-Raf	Skin inflammation	[60]
Carnosic acid		UVB	Phosphorylation	ERK, MEK	Photoaging	[61]
Plant polyphenols	Verbascoside	TGFα TNFα + IFNγ UVA + UVB LPS	Phosphorylation nuclear translocation	ERK, p65, Akt	Skin inflammation Skin cancer	[62]
	Resveratrol					
	Polydatin					
	Rutin					
	Quercetin					
Cyanidin-3-*O*-glucoside		UVB	Phosphorylation	P38, JNK, ERK, Akt	Epidermal cell apoptosis Skin cancer	[63]
1,8-Cineole		UVB	Phosphorylation (Y845)	P38, JNK1/2, ERK1/2 MEK1/2, B-Raf, C-Raf	Skin carcinogenesis	[44]
*Rhus javanica* extracts		UVB	Phosphorylation (T1068, T1045) EGFR activity	P38, JNK1/2, ERK1/2 MKK3/6, MKK4/7, MEK1/2, B-Raf, Akt, Src, PKD/PKCμ	Skin inflammation Photoaging	[64]
*Curcuma zedoaria* extracts		UVB	Phosphorylation (T1068, T1045)	P38, JNK1/2, ERK1/2 MKK3/6, MEK1/2, B-Raf, Akt, Src	Skin inflammation Photoaging	[65]
Curcumin		EGF	Phosphorylation (T1068) Surface plasmon resonance competition analysis			[66]
Silymarin		EGF	EGFR activity EGFR kinase activity			[67]
Epigallocatechin 3-Gallate		EGF	Phosphorylation of erbB1	ERK1/2		[68]
